# Subject-Wise Depression Screening from Eight-Channel Resting-State EEG Using Asymmetry-Aware Spectral Features and Connectivity Ablation

**DOI:** 10.3390/s26103065

**Published:** 2026-05-12

**Authors:** Hassan Ugail, Newton Howard, Ali Ahmed Elmahmudi, Zied Mnasri

**Affiliations:** 1 Centre for Visual Computing and Intelligent Systems, University of Bradford, Bradford BD7 1DP, UK; 2 The Howard Brain Sciences Foundation, Washington, DC 20001, USA

**Keywords:** electroencephalography, major depressive disorder, subject-wise evaluation, data leakage, spectral features, frontal alpha asymmetry, beta-band power, inter-channel coherence, Extra Trees, wearable EEG

## Abstract

Major depressive disorder remains difficult to diagnose objectively, as routine assessment is still largely dependent on clinical interview and rating scales. Resting-state electroencephalography (EEG) is an attractive complementary modality because it is non-invasive, low-cost, and compatible with wearable sensing, but many reported EEG classification results are weakened by segment-level leakage and unclear subject identity handling. This study evaluates whether depression can be distinguished from healthy controls using a compact eight-channel resting-state EEG configuration under a strictly leakage-free subject-wise protocol. Using a widely used public EEG dataset, we first corrected a previously overlooked subject-identity ambiguity by constructing a class-aware composite key, yielding 56 valid unique participants. We then applied ten repeated subject-wise holdout splits and compared five compact baselines spanning Extra Trees and a multi-layer perceptron on asymmetry-aware spectral features and three convolutional networks on raw signals, including the EEG-specific EEGNet and ShallowConvNet architectures. Uncertainty was quantified through 95% bootstrap confidence intervals of the mean across repeats. The best model, an Extra Trees classifier using eight-channel spectral and asymmetry features, achieved a mean balanced accuracy of 93.5% with a 95% bootstrap confidence interval of 89.6% to 96.8% and a mean area under the receiver operating characteristic curve of 98.6% with a 95% bootstrap confidence interval of 96.2% to 100.0%. A connectivity ablation showed that inter-channel coherence was informative in isolation but did not improve performance when naively fused with spectral features. A feature-selection ablation did not show evidence that the 90-dimensional spectral representation was dominated by noisy or uninformative dimensions under this evaluation protocol. These results support compact, subject-wise evaluated EEG screening pipelines while highlighting the importance of rigorous leakage control.

## 1. Introduction

Major depressive disorder (MDD) is a heterogeneous psychiatric condition associated with persistent low mood, anhedonia, cognitive dysfunction, and a range of somatic symptoms that together impose a substantial personal and societal burden. It affects an estimated 280 million people worldwide and remains a leading contributor to years lived with disability [1]. Despite this burden, routine diagnosis still depends primarily on clinical interview and rating instruments such as the Hamilton Depression Rating Scale [2]. Although clinically valuable, these approaches are inherently subjective, time-consuming, and difficult to scale for early or population-level screening. The lack of robust physiological biomarkers continues to limit objective assessment and contributes to delayed or missed diagnosis in real-world care pathways [3]. Recent artificial intelligence systems based on spoken language have shown promising agreement with established depression scales [4], but there is increasing interest in complementary physiological sensing modalities that may provide a more direct window into the underlying neurobiological state.

Among such modalities, resting-state electroencephalography (EEG) is particularly attractive. It is non-invasive, relatively inexpensive compared with neuroimaging, offers millisecond temporal resolution, and is becoming more widely available in compact wearable formats suitable for practical sensing applications [5]. Resting-state acquisition also avoids the need for active task participation, making it more tolerant of variability in effort, cognition, and symptom severity. A substantial literature has linked resting-state EEG features to depressive neurophysiology. Frontal alpha asymmetry, often interpreted as reflecting altered approach-related motivational processing, remains one of the most widely studied electrophysiological correlates of depression [6,7]. Increased beta-band activity in frontal and central regions has been associated with hyperarousal and ruminative processing [8], while theta-band abnormalities have been linked to impaired prefrontal control and affect regulation [9]. Beyond local spectral power, resting-state studies have also reported altered functional coupling, including reduced alpha-band coherence between frontal and parietal regions, suggesting disrupted long-range regulatory networks in MDD [10,11].

These neurophysiological observations have motivated a large and rapidly expanding body of literature on automated EEG-based depression detection. Reviews by Yasin et al. [12] and Rehman et al. [9] document a broad methodological spectrum, ranging from traditional machine learning pipelines based on engineered spectral features to deep learning approaches such as convolutional, recurrent, transformer, and distributed learning models [13], with transfer learning strategies enabling competitive performance on limited specialised datasets in adjacent domains [14]. Within the related literature, Lin et al. [15] showed that carefully engineered traditional models can remain highly competitive with end-to-end deep learning on resting-state EEG. Zandbagleh et al. [8] further highlighted the diagnostic relevance of periodic and aperiodic spectral structure, while Duta and Sultana [16] and Wang et al. [17] explored recent deep learning and multimodal fusion strategies. More broadly, Schirrmeister et al. [18] demonstrated that convolutional networks can learn directly from raw EEG, whereas Lotte et al. [19] and Craik et al. [20] reviewed common algorithmic choices and methodological challenges across EEG classification problems. Thus, these studies suggest that EEG contains discriminative information for depression screening, but they also show that reported performance depends strongly on evaluation design, feature representation, and cohort definition.

A central problem in this literature is that many EEG classification studies are evaluated in ways that are overly optimistic. Resting-state EEG is commonly segmented into multiple epochs per participant before feature extraction or model training. If train/test partitioning is then performed at the segment level rather than the participant level, segments from the same individual can appear in both sets. Because EEG contains substantial within-subject identity information, a classifier evaluated under this regime may learn person-specific patterns rather than disease-related signal, inflating apparent performance without reflecting true diagnostic generalisation. This issue has been explicitly identified as a barrier to clinical translation [3]. Related concerns arise from dataset heterogeneity, including inconsistencies in diagnostic definition, medication exposure, and population composition, all of which complicate cross-study comparison and external validity [21]. For depression-related EEG research, these are not minor technicalities. In fact, they directly affect whether a model’s reported accuracy can be interpreted as meaningful.

In the public EEG dataset examined in this study, this problem is compounded by a less obvious but critical subject-identity ambiguity. Raw subject identifiers are reused across diagnostic folders, such that labels like “S1” and “S7” appear in both the MDD and healthy control directories while referring to different individuals. Any pipeline that treats the raw identifier as globally unique will silently merge distinct participants across classes before evaluation even begins. If segment-level splitting is then applied, leakage occurs not only within individuals but also across the corrupted class roster, producing results that cannot be interpreted reliably. To the best of our knowledge, no published study using this dataset has simultaneously corrected this identity ambiguity, enforced repeated subject-wise evaluation, and performed a controlled ablation of spectral and connectivity representations under the corrected protocol.

This study addresses those issues directly. Our contribution is methodological and benchmarking-oriented rather than architectural. First, we correct the subject-identity ambiguity by constructing a class-aware composite key, yielding 56 valid unique participants for the final analysis. Second, we adopt a ten-repeat stratified subject-wise holdout protocol so that performance is characterised as a distribution rather than a single split-dependent point estimate. Third, we focus on an eight-electrode montage spanning frontal, central, and parietal sites, motivated both by prior neurophysiological evidence and by the practical requirements of wearable sensing hardware. Finally, we evaluate two related experimental questions, i.e., whether compact classical and deep models remain effective under leakage-free subject-wise testing, and whether inter-channel connectivity adds complementary information beyond asymmetry-aware spectral features. To answer the latter, we perform a systematic ablation comparing spectral features alone, connectivity features alone, and their naive early fusion.

The remainder of the paper is organised as follows. Section 2 describes the dataset correction, preprocessing assumptions, feature extraction pipeline, model configurations, and evaluation protocol. Section 3 reports the compact baseline results and the connectivity ablation. Section 4 interprets the findings in relation to depression neurophysiology, wearable sensing, and methodological rigour. Section 5 summarises the principal conclusions and outlines directions for future work.

## 2. Materials and Methods

### 2.1. Dataset and Subject Identity Correction

In this study, we have used the public resting-state EEG dataset released by Mumtaz et al. [22] and described by Mumtaz et al. [23], available on figshare (DOI https://doi.org/10.6084/m9.figshare.4244171.v2). The dataset contains recordings from individuals diagnosed with major depressive disorder (MDD) and healthy controls (HCs), sampled at 256 Hz with 19 electrodes placed according to the international 10–20 system. Recordings were acquired under eyes-open (EO), eyes-closed (EC), or both resting-state conditions, and recording durations differed across participants.

A critical issue in this dataset is that raw subject identifiers are reused across diagnostic folders. For example, identifiers such as “S1” and “S7” appear in both the MDD and HC directories while referring to different individuals. Any analysis that treats the raw identifier as globally unique will therefore merge distinct participants across classes before evaluation begins, making valid subject-wise splitting impossible. To avoid this, all downstream processing used the class-aware composite identifier defined in Equation (1), where label denotes the diagnostic class (MDD or H) and subject_id is the original folder-level identifier,

(1) subject_key:=label∥“_”∥subject_id.

For example, a patient record with identifier S1 becomes MDD_S1, while the healthy-control record sharing the same raw label becomes H_S1. Under this convention, MDD_S1 and H_S1 are treated as different participants throughout preprocessing, splitting, aggregation, and evaluation. After applying this correction and retaining only participants with usable preprocessed EEG segments, the final analysis cohort comprised 56 corrected unique subjects, of whom 31 belonged to the MDD group and 25 to the HC group. This subject identity correction is fundamental to the validity of the present study and is encoded in the released analysis pipeline.

The diagnostic labels in this dataset were assigned at the time of original data collection following standard clinical practice for psychiatric assessment. As described by Mumtaz et al. [23], MDD diagnosis was established by a qualified psychiatrist according to the *Diagnostic and Statistical Manual of Mental Disorders*, fourth edition, criteria, with depression severity quantified using the Beck Depression Inventory, second edition. Participants in the healthy control group had no history of psychiatric or neurological illness. The present study uses these labels as released and does not re-derive them from the EEG signal. We further note that the public release contains only MDD and healthy control labels. It does not include patients with anxiety, insomnia, bipolar disorder, or other psychiatric conditions that share overlapping neurophysiological features with depression. Differential diagnosis between depression and related disorders therefore lies outside the scope of this dataset and is discussed as a limitation in Section 4.5.

### 2.2. Channel Selection and Signal Processing

Although the source recordings contain 19 channels, the final analysis was restricted to eight electrodes: Fp1, Fp2, F3, F4, C3, C4, P3, and P4. This reduced selection was chosen for two reasons. First, frontal, central, and parietal regions are the sites most consistently implicated in depression-related spectral asymmetry, beta-band alterations, and disrupted long-range coupling [6,7,10]. Second, an eight-channel setup is consistent with compact wearable EEG hardware, making the proposed pipeline more relevant to practical sensing applications [5]. Temporal and occipital channels were excluded in order to focus on the most theoretically relevant sites while reducing susceptibility to ocular and muscular contamination.

Signal preprocessing was performed in a dedicated upstream pipeline, and the notebook used in the present paper consumes the resulting segment-level arrays. Raw EEG signals recorded at 256 Hz were bandpass filtered to remove slow drift and high-frequency noise, segmented into fixed-length resting-state epochs, screened for artefacts using amplitude-based rejection, and reduced to the eight selected channels. For participants with both EO and EC recordings, segments from both conditions were retained and pooled, while condition labels were preserved in the intermediate metadata. The total number of accepted artefact-free training segments across the ten repeated holdout draws ranged from 1573 to 1833, varying based on which subjects were assigned to the training partition in each repeat. The complete preprocessing implementation is provided in the upstream notebook (see Data Availability).

All model training was performed at the segment level, but all reported evaluations were performed at the subject level. Specifically, segment-level class probabilities were first computed for each test subject and then averaged across all segments belonging to that subject. The final subject prediction was obtained by thresholding the aggregated probability. This subject-level aggregation is central to the evaluation design because the scientific question is whether the system generalises across individuals rather than across segments extracted from the same recording.

To make leakage control fully transparent, we summarise here exactly which preprocessing operations are subject-independent and which are estimated from training data only. Bandpass filtering, segmentation into fixed-length epochs, and amplitude-based artefact rejection are applied per segment without reference to subject identity, class label, or split assignment, so they cannot transfer information across the train and test partitions. Median imputation of missing values, feature standardisation prior to the multi-layer perceptron, and per-channel z-score normalisation prior to the convolutional baselines were all fitted on training subjects only and reused unchanged for the held-out test subjects. For the feature-based pipelines, this was enforced automatically through the scikit-learn Pipeline interface, which fits all transformers on the training partition before applying them to test data. For the convolutional baselines, the per-channel mean and standard deviation are computed on the inner training split alone, after subject-wise removal of validation participants. Under this design, no statistic, parameter, or representation derived from any test subject is used during model fitting, which removes the implicit-leakage concern that arises when normalisation is fitted across the full dataset.

### 2.3. Feature Extraction

#### 2.3.1. Spectral Features

For each accepted segment and each selected channel, spectral power was estimated in five canonical frequency bands: delta (1–4 Hz), theta (4–8 Hz), alpha (8–12 Hz), beta (12–30 Hz), and gamma (30–40 Hz). Two complementary representations were then derived for each band. The first was *relative band power,* defined as band-specific power divided by the summed power across all five bands, thereby reducing sensitivity to inter-subject amplitude scaling. The second was *log-absolute band power,* obtained by applying the natural logarithm to absolute band power in order to compress skewed power distributions.

To encode hemispheric imbalance explicitly, inter-hemispheric asymmetry features were also computed for homologous left–right channel pairs: Fp1–Fp2, F3–F4, C3–C4, and P3–P4. These asymmetry terms were calculated as signed normalised differences between right- and left-hemisphere power. The final spectral representation, therefore, consisted of 80 per-channel power features (8 channels × 5 bands × 2 power representations) plus 10 asymmetry features (5 bands × 2 paired sites aggregated across homologous comparisons), yielding a **90-dimensional asymmetry-aware spectral feature vector**. This representation was designed to capture both absolute and lateralisation-related electrophysiological signatures of depression.

#### 2.3.2. Connectivity Features and Fusion

Functional connectivity was represented using magnitude-squared coherence, computed with the Welch method [24] through scipy.signal.coherence [25]. Coherence was evaluated for all 28 unique channel pairs generated from the eight-channel montage. For each pair, the mean coherence was extracted in the alpha (8–12 Hz) and beta (12–30 Hz) bands, producing a 56-dimensional connectivity feature vector per segment. These features were intended to capture pairwise linear synchronisation between frontal, central, and parietal regions.

For the fusion condition, the 90-dimensional spectral vector and the 56-dimensional coherence vectors were concatenated directly, resulting in a 146-dimensional fused representation. No dimensionality reduction or intermediate projection step was applied before concatenation. Consequently, the fusion results should be interpreted specifically as an evaluation of naive early fusion.

### 2.4. Classifiers

Three classifier families were considered across the two experiments. For feature-based models, data processing was implemented with scikit-learn [26]. Missing values, when present, were handled with median imputation. Feature scaling was applied for the MLP, but not required for the tree-based model.

The primary classifier was Extra Trees [27], an ensemble of highly randomised decision trees in which both the split variable and split threshold are randomly selected at each node. The model used 300 trees, no explicit maximum depth constraint, the Gini impurity criterion, and balanced class weighting. Extra Trees was selected as the main benchmark because it provides strong performance in tabular feature settings while also yielding directly interpretable Gini-based feature importance scores.

The second feature-based model was a multi-layer perceptron implemented with MLPClassifier. It comprised two hidden layers with 128 and 64 units, respectively, rectified linear unit activations, and an $L_{2}$ regularisation coefficient of $10^{-4}$, the Adam optimiser [28], a maximum of 200 iterations, and early stopping based on an internal 15% validation fraction. The MLP was evaluated on the same engineered feature spaces as Extra Trees, allowing comparison between a non-parametric ensemble and a compact gradient-based neural baseline.

The third model was a compact one-dimensional convolutional neural network operating directly on raw eight-channel EEG segments. The network was implemented in PyTorch [29]. It comprised three sequential temporal convolutional blocks. The first applies an 8-to-32 channel convolution with kernel size 7, followed by batch normalisation, ReLU, and max pooling. The second applies a 32-to-64 channel convolution with kernel size 5, again followed by batch normalisation, ReLU, and max pooling. The third applies a 64-to-128 channel convolution with kernel size 5, followed by batch normalisation, ReLU, and adaptive average pooling. The classification head used dropout, a fully connected layer with 64 hidden units, and a final linear output layer. Optimisation used binary cross-entropy with logits, Adam with a learning rate $10^{-3}$, a batch size of 64, and a maximum of 25 epochs. Early stopping with patience 5 was applied using a validation set constructed from 20% of the training subjects. To avoid leakage, per-channel normalisation statistics were estimated on training data only and reused unchanged for validation and test data.

To complement the compact 1D CNN with EEG-specific deep architectures, we also evaluated EEGNet [30] and ShallowConvNet [18], both implemented in PyTorch 1.2 under the same training protocol as the 1D CNN. EEGNet was configured with eight temporal filters in the first block, a depth multiplier of two for the depthwise spatial convolution, sixteen pointwise filters in the separable convolution of the second block, a temporal kernel length equal to half the sampling rate, and dropout of 0.5. ShallowConvNet was configured with forty temporal filters of length 25, forty spatial filters across the eight channels, mean pooling with a window of 75 samples and a stride of 15 samples, square activation followed by logarithmic compression, and dropout of 0.5. Both networks were trained with binary cross-entropy with logits, the Adam optimiser [28] at learning rate $10^{-3}$, batch size 64, and the same early-stopping criterion as the 1D CNN. In every repeat, EEGNet, ShallowConvNet, and the 1D CNN used the same outer subject-wise split, the same inner subject-wise validation partition, and the same per-channel z-score normalisation statistics estimated from training subjects.

The choice of these specific deep architectures is deliberate. EEGNet and ShallowConvNet are widely adopted EEG-tailored convolutional networks that are explicitly designed for small-sample EEG classification. They have well-characterised parameter counts and inductive biases, which make them appropriate references for whether EEG-specific deep learning can outperform a feature-based pipeline on a 56-subject cohort. We did not include transformer-based, graph-based, or Bayesian deep models. Such architectures typically require either substantially more training subjects, dedicated graph topologies, or auxiliary metadata that is not available in this public dataset. Including such models without the conditions under which they are designed to generalise would have produced weak baselines that obscure rather than clarify the comparison. These architectures are nevertheless useful directions for future work and are revisited in Section 4.5.

### 2.5. Evaluation Protocol and Experimental Design

All experiments used a ten-repeat subject-wise holdout protocol. In each repeat, the 56 corrected subjects were partitioned into approximately 80% training subjects (∼45 individuals) and 20% test subjects (∼13 individuals), while preserving class balance as closely as possible. On average, each test fold contained about seven MDD participants and six healthy controls. The random seed was initialised at 42 and incremented across repeats to ensure deterministic reproducibility while still sampling multiple independent holdout draws. For the convolutional baselines, an additional 20% of the training subjects were set aside for early-stopping validation. The decision to use ten repeats was a tractability choice. With 56 unique subjects, a single subject-wise holdout draw provides only one operating point and is highly sensitive to which subjects fall into the test partition. Repeating the draw ten times produces a sample of ten subject-wise operating points, which is sufficient to characterise dispersion while keeping the total training cost manageable for the deep baselines. We do not claim that ten repeats prove asymptotic stability. Rather, the dispersion across these ten repeats is reported explicitly, both as standard deviation and as a 95% bootstrap confidence interval of the mean, so that the reader can judge sampling variability directly.

Performance was assessed at the subject-level using balanced accuracy, area under the receiver operating characteristic curve (AUROC), sensitivity, specificity, precision, F1 score, and Matthews correlation coefficient (MCC) [31]. All reported results are mean across the ten repeats accompanied by a 95% confidence interval of the mean estimated through 10,000 percentile bootstrap resamples of the per-repeat values, in addition to the per-repeat standard deviation. These bootstrap intervals are intended as descriptive uncertainty summaries over the ten repeated holdout results rather than as formal population-level confidence bounds, given the small number of repeats relative to the bootstrap resample budget. Because each test split contains only about 13 individuals, even a single additional subject-level misclassification can change balanced accuracy by roughly 8 to 12 percentage points. The reported standard deviations should therefore be interpreted as a direct consequence of honest repeated subject-wise evaluation rather than as an instability specific to one model family.

Three experiments were performed. Experiment 1 compared five compact classifiers on the eight-channel configuration. Two operated on the asymmetry-aware spectral feature vector, namely Extra Trees and the multi-layer perceptron. Three operated directly on the raw eight-channel segments, namely the compact 1D CNN, EEGNet, and ShallowConvNet. This experiment establishes the main leakage-free compact baselines against both engineered and learned representations. Experiment 2 examined the contribution of functional connectivity by comparing Extra Trees trained on spectral features only, connectivity features only, and fused spectral and connectivity features, together with a multi-layer perceptron trained on the fused representation. This design isolates whether coherence contributes additional discriminative value once spectral information is already available. Experiment 3 probed whether the 90-dimensional spectral representation is over-parameterised. For each repeat, we fitted Extra Trees on the full spectral representation, ranked features by Gini importance, then refitted and re-evaluated Extra Trees on the top *K* features for K∈{5,10,15,20,30,50,70,90} under the same subject-wise test split. Because the importance ranking is recomputed inside every repeat from training data only, this ablation does not introduce additional leakage.

## 3. Results

### 3.1. Compact Eight-Channel Baselines

Table 1 summarises the results of the ten repeated subject-wise holdout evaluations for the five compact baseline models. For each metric we report the mean across repeats together with the standard deviation across repeats. For balanced accuracy and AUROC we also report the 95% bootstrap confidence interval of the mean. On average, each test split contained approximately 13 subjects, including about seven participants with MDD and six healthy controls. This small test-fold size is important when interpreting the dispersion of the results. Under subject-wise evaluation, a single additional subject-level error can change the balanced accuracy by roughly 8 to 12 percentage points.

Among the five baseline models, the Extra Trees classifier trained on asymmetry-aware spectral features achieved the strongest overall performance. It obtained a mean balanced accuracy of 93.5% with a 95% bootstrap confidence interval of 89.6% to 96.8%, and a mean AUROC of 98.6% with a 95% bootstrap confidence interval of 96.2% to 100.0%. The sensitivity for the MDD class reached 98.6% with a standard deviation of 4.5%, indicating that the model correctly identified nearly all depressed participants across repeated test splits, while the specificity for healthy controls was 88.3% with a standard deviation of 11.2%. The corresponding multi-layer perceptron, evaluated on the same spectral feature representation, also performed strongly, with a mean balanced accuracy of 90.2% (95% CI 86.1% to 95.0%) and a mean AUROC of 97.6% (95% CI 95.0% to 100.0%). This indicates that the engineered spectral representation is discriminative enough to support both tree-based and neural classifiers.

**Figure 1:**
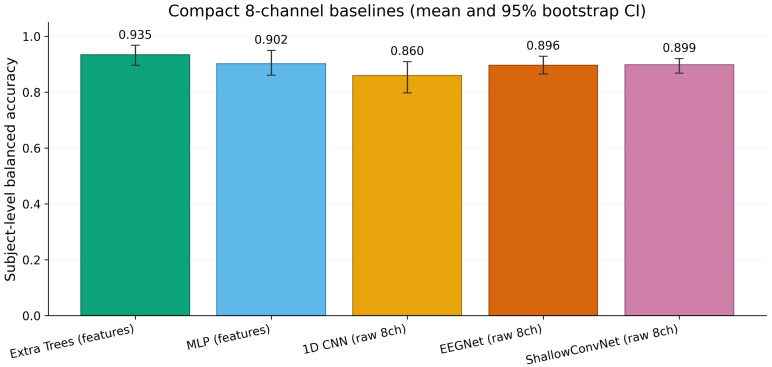
Performance comparison of the five compact eight-channel baseline classifiers under ten repeated subject-wise holdout splits. The Extra Trees model trained on asymmetry-aware spectral features achieved the highest mean balanced accuracy. Among the raw-signal deep baselines, ShallowConvNet was the strongest, followed by EEGNet and the compact 1D CNN. Error bars denote the 95% bootstrap confidence interval of the mean estimated from 10,000 percentile bootstrap resamples of the ten per-repeat values.

Among the raw-signal deep baselines, ShallowConvNet achieved the highest mean balanced accuracy at 90.7% (95% CI 87.1% to 93.8%) and a mean AUROC of 98.3% (95% CI 96.0% to 100.0%). EEGNet followed at 89.6% balanced accuracy (95% CI 86.6% to 92.9%) and a mean AUROC of 97.1% (95% CI 95.0% to 99.0%). The compact 1D CNN obtained the lowest mean balanced accuracy at 86.0% (95% CI 79.8% to 91.0%) and a mean AUROC of 97.1% (95% CI 95.0% to 99.0%). Although ShallowConvNet and EEGNet are EEG-tailored convolutional architectures, neither exceeded the spectral Extra Trees baseline, and their confidence intervals overlap substantially with the multi-layer perceptron operating on the same spectral feature space. In practical terms, the feature-based Extra Trees model therefore provides the most stable and interpretable compact benchmark for this dataset under leakage-free subject-wise testing. Figure 1 visualises the same comparison.

### 3.2. Connectivity Ablation

The connectivity ablation results are reported in Table 2. The principal finding is that the spectral representation already captures most of the discriminative signal available in this eight-channel setting. Extra Trees trained on spectral features alone achieved a mean balanced accuracy of 93.5% (95% CI 89.6% to 96.8%) and a mean AUROC of 98.6% (95% CI 96.2% to 100.0%). Importantly, the corresponding fusion model, in which spectral and coherence features were concatenated before classification, achieved the same mean balanced accuracy and the same mean AUROC. Although the fusion model showed slightly lower variability in balanced accuracy (5.0% versus 6.4% standard deviation), it did not improve any of the central point estimates and its 95% bootstrap confidence interval (90.5% to 96.7%) overlapped almost completely with the spectral baseline.

The connectivity-only model remained clearly above chance, with a mean balanced accuracy of 79.6% (95% CI 75.5% to 83.9%) and a mean AUROC of 90.7% (95% CI 87.6% to 94.0%). This confirms that inter-channel coherence carries meaningful information about depressive brain-state differences. However, its performance remained well below the spectral baseline, with an approximate gap of 13.9 percentage points in balanced accuracy and non-overlapping 95% confidence intervals. The fused multi-layer perceptron also did not improve on the feature-only multi-layer perceptron baseline from Experiment 1, suggesting that the higher-dimensional fused space introduces additional modelling difficulty without yielding complementary predictive gain.

**Figure 2:**
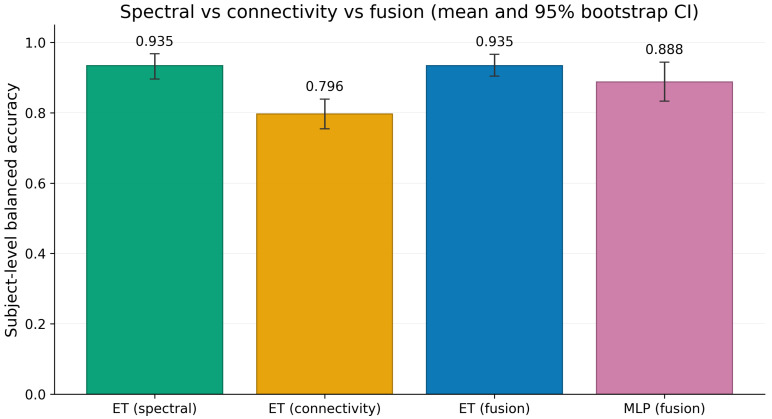
Connectivity ablation across four model configurations under ten repeated subject-wise holdout splits. Spectral-only Extra Trees and fused Extra Trees achieved the same mean balanced accuracy with overlapping 95% confidence intervals, indicating that naive early fusion had no practical benefit. The connectivity-only model remained above chance but substantially below the spectral baseline, with non-overlapping confidence intervals. The fused multi-layer perceptron also underperformed the best spectral model. Error bars denote the 95% bootstrap confidence interval of the mean.

These results indicate that coherence is informative in isolation but does not add useful marginal information when combined with spectral features through naive early fusion. Figure 2 presents the same result graphically.

### 3.3. Spectral Feature-Selection Ablation

To probe whether the 90-dimensional asymmetry-aware spectral representation is over-parameterised, we conducted a top-*K* feature-selection ablation under the same repeated subject-wise holdout protocol. In each repeat, Extra Trees was first fitted on the full 90-feature representation using only training subjects, the resulting Gini importance ranking was computed, and Extra Trees was then refitted and re-evaluated on the top *K* features for K∈{5,10,15,20,30,50,70,90}. The ranking is recomputed inside every repeat from training data only, so the ablation does not introduce additional leakage.

Table 3 reports the mean balanced accuracy and AUROC for each *K*, accompanied by the standard deviation across repeats and the 95% bootstrap confidence interval of the mean. Across the entire grid, the mean balanced accuracy ranged from 87.9% at K=10 to 94.3% at K=90, with an interquartile span of approximately three percentage points between adjacent operating points. The 95% confidence intervals for K≥5 all overlap substantially with the full 90-feature operating point, and no value of *K* smaller than 90 produced a higher mean than the full representation. Equivalently, restricting Extra Trees to as few as five features still preserved a mean balanced accuracy of 90.8%, which is well above any of the deep baselines reported in Section 3.1.

We note that the K=90 row of Table 3 differs slightly from the spectral Extra Trees row of Table 1, despite both using the full 90-feature representation. In the feature-selection ablation, Extra Trees is first fitted to derive the Gini importance ranking, then refitted on the importance-sorted column order. This column order differs from the alphabetical column order used in Experiment 1. Because Extra Trees randomises the candidate-feature subset at each split based on column index, the refit takes a slightly different random path even with the same random seed, producing a small numerical difference that is well within sampling variability. The two values fall within each other’s 95% bootstrap confidence intervals.

**Figure 3:**
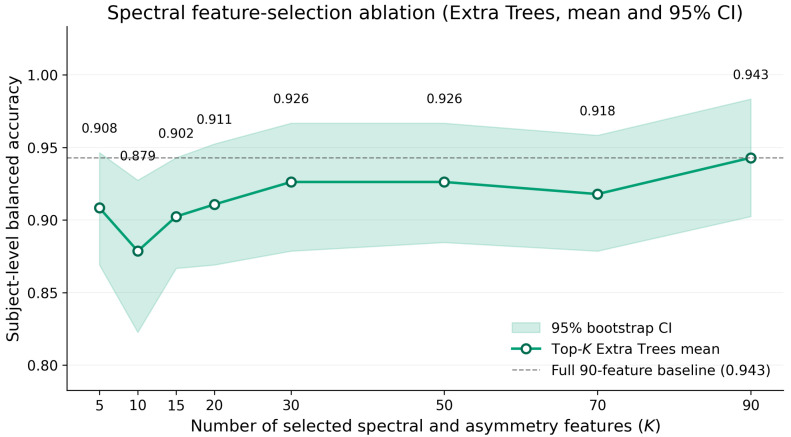
Spectral feature-selection ablation. Subject-level balanced accuracy is reported as the mean across the ten repeated subject-wise holdout splits, with the shaded band denoting the 95% bootstrap confidence interval of the mean estimated from 10,000 percentile bootstrap resamples. The horizontal dashed line marks the full 90-feature operating point. No reduced feature set exceeded the full representation, while restricting Extra Trees to as few as five features still preserved a mean balanced accuracy of 90.8%.

The practical interpretation is twofold. First, the feature-selection ablation does not show evidence that the 90-dimensional spectral representation is harmful or dominated by noisy or uninformative dimensions under this evaluation protocol. If the representation were dominated by such dimensions, performance at smaller *K* would systematically exceed performance at K=90, which is not observed. Second, a small subset of features carries most of the discriminative signal. The Gini-ranked top five already reach approximately 91% mean balanced accuracy, which suggests that further channel and feature reduction is feasible for highly compact deployment scenarios. Figure 3 shows the same result graphically.

### 3.4. Feature Importance Analysis

**Figure 4:**
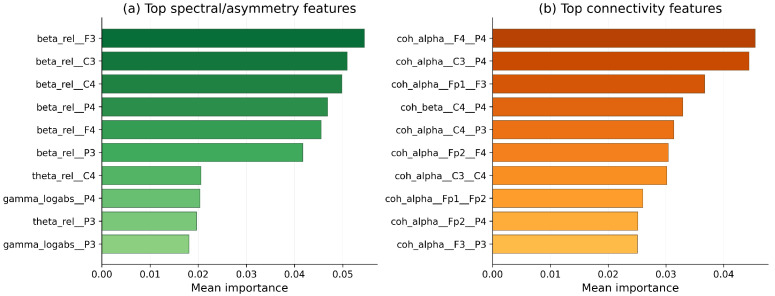
Mean Gini importance scores averaged across ten repeated subject-wise holdout splits for the Extra Trees models. The left panel (**a**) shows the spectral and asymmetry model, where relative beta-band power at frontal, central, and parietal sites dominates. The right panel (**b**) shows the connectivity-only model, where alpha-band fronto-parietal and fronto-frontal coherence features lead. Both panels show that spectral and connectivity representations carry physiologically meaningful signals, even though naive early fusion did not improve the overall classification performance.

Feature-importance analysis was performed using the mean Gini importance scores from the Extra Trees models, averaged across the ten holdout repeats. Figure 4 shows the results in two panels: the left panel shows the top spectral and asymmetry features from the feature-based Extra Trees model, while the right panel shows the top connectivity features from the coherence-only model.

A consistent pattern emerged in the spectral ranking. Relative beta-band power at frontal, central, and parietal electrodes dominated the top positions, with beta_rel_F3, beta_rel_C3, beta_rel_C4, beta_rel_P4, and beta_rel_F4 all appearing prominently. Secondary contributions came from theta-band relative power and log-absolute gamma power, especially at central and parietal sites. The prominence of bilateral frontal and central beta features suggests that the classifier exploits both elevated beta activity and its hemispheric distribution, which is consistent with prior work linking depression to altered frontal asymmetry and heightened beta-related hyperarousal [6,7].

The connectivity ranking was led primarily by alpha-band coherence between frontal and parietal electrodes, including pairs such as F4–P4, C3–P4, Fp1–F3, and C4–P3. Beta-band central–parietal coherence also appeared among the top contributors. This pattern is compatible with previous reports of altered long-range frontal–parietal coupling in depression [10,11]. However, when spectral and connectivity features were fused, the overall classification performance did not improve, reinforcing the interpretation that the coherence features are informative but not sufficiently complementary to the spectral representation under simple concatenation.

## 4. Discussion

### 4.1. Methodological Significance of the Corrected Subject-Wise Protocol

The central contribution of this study is methodological before it is algorithmic. The headline performance of the best model is important, but the more consequential result is that these numbers were obtained under an evaluation design intended to reflect genuine subject-level generalisation. In this dataset, two distinct forms of leakage can arise if care is not taken. First, subject identifiers are reused across diagnostic folders, and second, EEG segments can easily be split at the epoch level rather than at the participant level. Either issue is sufficient to inflate performance estimates. Together, they make the reported results difficult to interpret. By introducing a class-aware composite subject key and enforcing repeated subject-wise holdout evaluation, the present study addresses both problems directly.

This point is not merely procedural. In EEG classification, within-subject signal structure is often highly stable across segments, and models can exploit subject identity even when the intended task is disease classification. Reported performance under segment-level splitting may therefore reflect recognition of individual-specific recording characteristics rather than the detection of depression-related neurophysiology. This study was designed to avoid that failure mode. The resulting figures, 93.5% mean balanced accuracy and 98.6% mean AUROC for the strongest model, should therefore be interpreted not as spectacularly high outcomes in isolation, but as plausible and defensible estimates for a small public dataset evaluated under stricter conditions than are often used in the literature.

The repeated holdout design also reveals a point that single-split evaluations obscure. Uncertainty at this cohort size is substantial. With approximately 13 test subjects per repeat, a single additional error changes balanced accuracy by several percentage points. The observed standard deviations are therefore not a weakness of the analysis but a more honest reflection of sampling variability under subject-wise evaluation. From a benchmarking perspective, this is valuable. It shifts the emphasis away from isolated best-case numbers and toward performance distributions, which are more informative for comparing models and for assessing whether apparent gains are likely to be meaningful.

### 4.2. Why Spectral and Asymmetry Features Dominated

The strongest-performing model in this study was the Extra Trees classifier trained on asymmetry-aware spectral features from only eight electrodes. This result is consistent with a substantial body of prior work suggesting that resting-state depression-related EEG differences are often expressed more clearly in relatively low-dimensional power-based descriptors than in more complex representations. In particular, the feature-importance analysis showed that relative beta-band power at frontal and central electrodes carried much of the discriminative weight, with additional contributions from parietal beta, theta-band features, and selected higher-frequency power terms.

This ranking is neurophysiologically plausible. Elevated beta activity has been linked to hyperarousal, sustained internal mentation, and ruminative cognitive style, all of which are relevant to the clinical phenotype of major depressive disorder. The fact that both left and right frontal and central electrodes appeared among the highest-ranked predictors suggests that the model was not relying on a single local abnormality but on a distributed pattern combining absolute power and hemispheric imbalance. This is compatible with the broader frontal asymmetry literature, but the present results also suggest that useful signal extends beyond a narrowly defined alpha asymmetry formulation. In this sense, the spectral representation used here may be understood as a pragmatic extension of the asymmetry framework. It preserves interpretable lateralisation cues while allowing the classifier to exploit additional frequency- and region-specific effects. Tree-based importance rankings provide one perspective on model behaviour, and complementary multi-method interpretability strategies that combine model-internal attribution with independent explanation tools could strengthen biological interpretation in future work [32].

The relative strength of the engineered spectral representation compared with the raw-signal deep baselines is also informative. The 1D CNN achieved a competitive performance, which indicates that meaningful class-related information is present in the raw segments themselves. ShallowConvNet and EEGNet, both of which are designed specifically for EEG classification, also reached competitive but lower accuracy than the spectral Extra Trees baseline, with overlapping confidence intervals among themselves and with the multi-layer perceptron operating on the same spectral features. None of the three deep architectures exceeded the spectral baseline, and the 1D CNN showed the greatest inter-repeat variability. For a cohort of this size, this is not surprising. Deep models trained on raw EEG often require larger and more diverse training sets to consistently outperform well-designed feature-based pipelines. In addition, the feature-selection ablation in Section 3.3 does not show evidence that the 90-dimensional spectral representation is harmful or dominated by noisy dimensions under this evaluation protocol. Performance does not improve when Extra Trees is restricted to smaller importance-ranked subsets, and even five features preserve mean balanced accuracy at approximately 91%. Thus, these results support a conservative conclusion that for compact resting-state EEG depression screening under limited-sample conditions, interpretable spectral and asymmetry-aware features remain a strong and practical first-line representation.

### 4.3. Connectivity Is Informative, but Naive Fusion Is Not

The connectivity ablation adds an important layer to the interpretation of the results. The coherence-only model performed clearly above chance, demonstrating that inter-channel coupling contains genuine information relevant to depression classification. Moreover, the most prominent connectivity features were concentrated in frontal–parietal and frontal–frontal alpha coherence, together with selected beta coherence terms. This pattern fits well with prior reports of altered long-range functional coordination in depression, particularly that involving fronto-parietal regulatory networks.

At the same time, the fusion experiment showed that simply concatenating coherence features with spectral features did not improve performance beyond the spectral baseline. This is a negative result, but it is a meaningful one. It suggests that the relationship between spectral content and pairwise coherence in this dataset is not strongly complementary under simple early fusion. A likely explanation is partial redundancy, i.e., when two channels exhibit elevated or coordinated activity in a given band, the associated coherence measure may reflect information that is already partly captured by the power terms themselves. Accordingly, connectivity features can still be informative in isolation while failing to contribute additional discriminative value once a strong spectral representation is already present.

This finding has practical implications. First, it argues against assuming that increasing feature dimensionality will necessarily improve depression classification performance in compact EEG settings. Second, it suggests that future multimodal EEG representations should not rely on concatenation alone if the goal is to exploit complementary information across feature families. More structured approaches, including directed connectivity measures, graph-based representations, source-level coupling metrics, or learned fusion mechanisms, may be required if connectivity is to contribute additional value beyond spectral information. The present results, therefore, do not imply that connectivity is unimportant. Rather, they indicate that the particular fusion strategy tested here is insufficient to unlock its marginal utility.

### 4.4. Implications for Compact Wearable EEG Systems

A notable practical aspect of this study is the restriction to an eight-electrode montage. This was motivated by both neurophysiological relevance and sensing feasibility. Frontal, central, and parietal sites are frequently implicated in depression-related EEG findings, and they are also among the most accessible positions in compact or wearable systems. Achieving strong subject-wise performance with only eight electrodes, therefore, supports the idea that useful depression-related EEG screening pipelines need not depend on dense clinical caps.

This does not mean that the present system is ready for deployment, but it does suggest that hardware-efficient designs are realistic targets. The feature-importance results are particularly useful in this context because they point toward a smaller subset of electrodes, especially frontal and central sites, as carrying a substantial proportion of the useful signal. That observation could inform future studies on further channel reduction, headset design, or adaptive sensor placement. In addition, the computational primitives used here, namely spectral estimation and coherence via standard signal-processing routines, are considerably lighter than many end-to-end deep architectures, which offers an advantage for portable or embedded sensing workflows. Robustness to imperfect or degraded acquisition is also a recurring concern in compact deployment settings, mirroring observations from other application domains where deep models must operate under input noise or partial information [33,34]. In wearable EEG specifically, motion artefacts, dry-electrode contact variability, and ambient electrical noise can all degrade signal quality compared with clinical-grade acquisition, and the present feature-based pipeline may offer a more predictable degradation profile than a high-capacity raw-signal model retrained from scratch on small clinical cohorts.

We note that the value of the present work lies not only in classification accuracy but also in showing that a leakage-aware, interpretable, and wearable-compatible pipeline can remain competitive. In that sense, the study contributes to the design space of practical physiological sensing systems as much as to the EEG depression literature itself.

### 4.5. Limitations and Future Directions

Several limitations must be acknowledged. First, the study is based on a single public dataset and a final analysable cohort of 56 subjects. Although repeated subject-wise holdout evaluation provides a more realistic estimate than segment-level splitting, the test sets remain small, and the resulting uncertainty is non-negligible. The decision to use exactly ten repeats was a tractability choice rather than a derivation from a formal statistical reliability criterion. We therefore report uncertainty explicitly using both per-repeat standard deviation and the 95% bootstrap confidence interval of the mean, so that the reader can judge sampling variability directly rather than relying on a single point estimate. External validation on independent cohorts is essential before any claim of broader clinical usefulness can be justified, and we note that public EEG depression datasets recorded under different protocols and equipment are not directly interchangeable with the present cohort [21].

Second, the available metadata is limited. Information such as medication status, illness duration, episode severity, comorbid conditions, and treatment history is insufficient in the public release. As a result, it is not possible to determine whether the classifier is responding specifically to depressive neurophysiology, to medication effects, or to other correlated factors. This is a common limitation in public psychiatric EEG datasets, but it constrains the biological interpretation of any classification result.

Third, the public release used in this study contains only patients with major depressive disorder and healthy controls. It does not include patients with anxiety, insomnia, or bipolar disorder, all of which can share overlapping resting-state EEG features with depression. The present pipeline therefore demonstrates discrimination of MDD from healthy controls but cannot speak to differential diagnosis between depression and other psychiatric or sleep-related disorders. This is a clinically important question, and it motivates future work using multi-condition cohorts that include comparator psychiatric groups in addition to healthy controls. Fourth, the connectivity analysis was intentionally simple. Only magnitude-squared coherence in the alpha and beta bands was examined, and fusion was implemented through direct feature concatenation. This was appropriate for a controlled ablation, but it leaves several richer alternatives unexplored, including phase-based coupling, effective connectivity, graph summary measures, source-space connectivity, and learned cross-feature fusion. The present results should therefore be interpreted as evidence against naive early fusion in this setting, not as a general rejection of connectivity-aware modelling.

Fifth, all reported results are from offline analysis under a single acquisition protocol. Online clinical deployment introduces engineering and usability factors that are not captured here, including inter-subject and intra-subject electrode placement variability, hardware-specific noise characteristics, motion artefacts in everyday environments, and the trade-off between dry sensor convenience and signal quality. The computational primitives used in the present pipeline are deliberately light, and the eight-channel montage maps naturally to compact wearable hardware, so the proposed approach is in principle compatible with on-device inference. However, the magnitude of any accuracy or sensitivity loss when transferring from clinical-grade laboratory recordings to wearable acquisition can only be estimated through dedicated on-hardware validation studies, which are beyond the scope of this paper. Finally, although the paper emphasises rigorous evaluation, full reproducibility still depends on the upstream preprocessing stage. Future public releases should ensure that the full pipeline, from raw EEG ingestion through accepted segment generation to final model evaluation, is documented in a single transparent workflow. That is especially important for datasets in which identity handling, segmentation strategy, and artefact rejection can substantially alter the apparent performance of the final classifier.

Several promising research directions follow naturally from these limitations. Bayesian classifiers and Bayesian deep models would allow principled uncertainty quantification at the level of individual subject predictions rather than only at the level of aggregated metrics, which is particularly valuable in a screening setting where the cost of false reassurance differs from the cost of false alarm. Attention-based and transformer architectures, including federated and split-learning variants [13], may exploit longer-range dependencies in resting-state EEG when sufficiently large training cohorts become available. Graph neural networks defined over the eight-channel coherence graph could integrate the connectivity ablation findings with learned message-passing representations and may unlock the marginal value of connectivity that naive concatenation did not. Each of these directions is more demanding in terms of cohort size and metadata than the present 56-subject public release supports. Within that practical constraint, the present study offers a defensible, leakage-controlled benchmark and a methodological foundation based on which more advanced models can be evaluated fairly. Thus, these limitations point to a clear future agenda that includes larger external validation studies, more detailed metadata-aware analyses, multi-condition cohorts that enable differential diagnosis, dedicated on-device evaluations of wearable EEG sensing, and more principled connectivity integration strategies.

## 5. Conclusions

In this study, we have examined whether people with major depressive disorder can be distinguished from healthy controls using a compact eight-channel resting-state EEG configuration when evaluation is performed under a genuinely subject-wise, leakage-free protocol. The results support three main conclusions.

First, valid benchmarking on this public dataset requires explicit correction of subject identity. Because raw subject identifiers are reused across diagnostic folders, any pipeline that groups records by the original identifier risks silently merging different individuals across classes. When such ambiguity is combined with segment-level train/test splitting, reported performance can no longer be interpreted as a meaningful estimate of subject-level generalisation. By introducing a class-aware composite subject key and enforcing repeated subject-wise holdout evaluation, this work establishes a more rigorous and transparent benchmark for future studies using the same dataset.

Second, asymmetry-aware spectral features from only eight electrodes provide a strong and practical representation for depression classification in this cohort. The best-performing model, an Extra Trees classifier, achieved a mean balanced accuracy of 93.5% (95% bootstrap CI 89.6% to 96.8%) and a mean AUROC of 98.6% (95% bootstrap CI 96.2% to 100.0%) across ten repeated subject-wise splits. A compact raw-signal 1D CNN, EEGNet, and ShallowConvNet were also evaluated under the same protocol. All three achieved a competitive performance, but none exceeded the feature-based baseline, and the 1D CNN showed the highest inter-repeat variability. A complementary feature-selection ablation showed no evidence that the 90-dimensional spectral representation is harmful or dominated by noisy dimensions, since no smaller importance-ranked subset improved on the full set. These findings suggest that, under limited-sample conditions, well-designed spectral and asymmetry features remain a highly effective and interpretable choice.

Third, functional connectivity carries relevant information, but its value depends on how it is incorporated. Coherence-based connectivity features alone performed better than chance, indicating that altered inter-regional coupling contributes to the depressive EEG signature. However, simple early fusion of spectral and connectivity features did not improve over the spectral baseline, implying that naive concatenation is insufficient to extract complementary benefit from the two representations in this setting.

Therefore, these results show that compact, wearable-compatible EEG configurations can support meaningful subject-wise depression screening benchmarks when the evaluation is designed carefully. Hence, this study contributes both a practical eight-channel reference pipeline and a methodological warning about leakage in public EEG depression datasets. Future work should validate these findings on larger and independent cohorts and examine whether the electrode set can be reduced further without substantial loss of performance, and explore more principled strategies for integrating spectral and connectivity information.

## Figures and Tables

**Table 1:** Compact eight-channel baseline performance across ten repeated subject-wise holdout splits. For balanced accuracy (BA) and AUROC, the table reports the mean followed by the standard deviation across repeats and the 95% bootstrap confidence interval of the mean in square brackets. For sensitivity (Sen.), specificity (Spe.), precision (Pre.), F1 score, and MCC, the table reports mean and standard deviation. MCC is dimensionless. All other metrics are percentages.

Model	BA (%)	AUROC (%)	Sen. (%)	Spe. (%)	Pre. (%)	F1 (%)	MCC
Extra Trees (spectral)	93.5±6.4 [89.6, 96.8]	98.6±3.8 [96.2, 100.0]	98.6±4.5	88.3±11.2	91.3±8.0	94.7±5.3	0.883±0.116
ShallowConvNet (raw)	90.7±5.6 [87.1, 93.8]	98.3±3.7 [96.0, 100.0]	91.4±13.8	90.0±8.6	92.3±6.6	91.0±7.3	0.829±0.104
MLP (spectral)	90.2±7.6 [86.1, 95.0]	97.6±4.2 [95.0, 100.0]	97.1±6.0	83.3±15.7	88.4±10.5	92.1±5.9	0.828±0.132
EEGNet (raw)	89.6±5.3 [86.6, 92.9]	97.1±3.5 [95.0, 99.0]	94.3±7.4	85.0±9.5	88.5±6.7	91.0±4.7	0.806±0.104
1D CNN (raw)	86.0±9.7 [79.8, 91.0]	97.1±3.7 [95.0, 99.0]	88.6±17.6	83.3±11.1	86.4±8.9	86.6±12.1	0.736±0.192

**Table 2:** Connectivity ablation performance across ten repeated subject-wise holdout splits. For balanced accuracy (BA) and AUROC, the table reports the mean followed by the standard deviation across repeats and the 95% bootstrap confidence interval of the mean in square brackets. For sensitivity (Sen.), specificity (Spe.), precision (Pre.), F1 score, and MCC, the table reports mean and standard deviation. The Extra Trees spectral-only condition is repeated from Experiment 1 for reference. MCC is dimensionless. All other metrics are percentages.

Model	BA (%)	AUROC (%)	Sen. (%)	Spe. (%)	Pre. (%)	F1 (%)	MCC
ET (spectral only)	93.5±6.4 [89.6, 96.8]	98.6±3.8 [96.2, 100.0]	98.6±4.5	88.3±11.2	91.3±8.0	94.7±5.3	0.883±0.116
ET (connectivity only)	79.6±7.2 [75.5, 83.9]	90.7±5.5 [87.6, 94.0]	84.3±10.5	75.0±19.6	81.9±11.4	82.0±5.1	0.617±0.125
ET (fusion)	93.5±5.0 [90.5, 96.7]	98.6±3.8 [96.2, 100.0]	98.6±4.5	88.3±8.1	91.1±6.2	94.6±4.4	0.881±0.096
MLP (fusion)	88.8±9.3 [83.3, 94.4]	97.1±4.2 [94.5, 99.5]	94.3±7.4	83.3±13.6	87.4±9.9	90.6±7.9	0.788±0.181

**Table 3:** Spectral feature-selection ablation across ten repeated subject-wise holdout splits. Extra Trees was refitted on the top *K* features by Gini importance, ranked inside each repeat using training data only. Balanced accuracy (BA) and AUROC are reported as the mean and standard deviation across repeats, with the 95% bootstrap confidence interval of the mean in square brackets. All values are percentages.

*K*	Features Used	BA (%)	AUROC (%)
5	5	90.8±6.9 [86.9, 94.6]	96.2±5.9 [92.4, 98.8]
10	10	87.9±8.8 [82.3, 92.7]	97.4±4.9 [94.0, 100.0]
15	15	90.2±6.5 [86.7, 94.3]	98.1±3.9 [95.5, 100.0]
20	20	91.1±7.2 [86.9, 95.2]	96.4±5.4 [93.1, 99.5]
30	30	92.6±8.0 [87.9, 96.7]	97.9±4.5 [95.0, 100.0]
50	50	92.6±7.1 [88.5, 96.7]	98.1±4.5 [95.2, 100.0]
70	70	91.8±6.7 [87.9, 95.8]	98.1±4.5 [95.2, 100.0]
90	90	94.3±6.7 [90.2, 98.3]	98.3±3.7 [96.0, 100.0]

## Data Availability

The EEG dataset analysed in this study is publicly available on figshare, DOI https://doi.org/10.6084/m9.figshare.4244171.v2. The code and data generated during the experimentation and analysis are publicly available at https://github.com/ugail/EEG_Based_Depression_Screening (accessed on 9 May 2026).
